# Microbiological profiling and the demonstration of *in vitro* anti-bacterial traits of the major oral herbal medicines used in Dhaka Metropolis

**DOI:** 10.1186/2193-1801-3-739

**Published:** 2014-12-15

**Authors:** Marufa Sharmin, Ifra Tun Nur, Mrityunjoy Acharjee, Saurab Kishore Munshi, Rashed Noor

**Affiliations:** Department of Microbiology, Stamford University Bangladesh, 51 Siddeswari Road, Dhaka, 1217 Bangladesh

**Keywords:** Drug resistance, In vitro anti-bacterial activity, Oral herbal medicines, Pathogens, Public health, Spoilage microorganisms

## Abstract

Present study attempted to assess the level of microbiological contamination in oral herbal medicines, frequently used for medications, through conventional cultural and biochemical tests along with the antibiogram of the isolates. Moreover, the anti-bacterial potential of the herbal medicines was also aimed to be checked by the agar well diffusion method and minimum inhibitory concentration (MIC) assay. Out of 10 categories of liquid oral herbal medicine samples (n = 50) studied, all were found to be contaminated with bacteria (10^3^-10^5^ cfu/mL), specifically with *Staphylococcus* spp. in 8 samples; while 2 samples harbored *Klebsiella* spp. Fungal presence was observed only in one sample. Study of antibiogram revealed *Klebsiella* spp. to be strongly resistant against penicillin G and erythromycin, whereas *S. aureus* possessed 80% sensitivity. The in vitro anti-bacterial activity was observed in 7 samples. Of them, one sample was found to exhibit the activity against almost all the test bacteria and another was found effective against 5 out of 8 test bacteria. Five samples showed the activity within a minor range while 3 samples were devoid of such trait. Samples 2 and 4 were found to stall the bacterial growth below 10 mg/mL of concentration in MIC test. Overall, the prevalence of specific pathogens was not so significant in the samples studied as well as only one drug-resistant isolate was identified. Besides, the anti-bacterial trait of 5 samples indicated that most of herbal medicines might be considered effective for medication.

**Electronic supplementary material:**

The online version of this article (doi:10.1186/2193-1801-3-739) contains supplementary material, which is available to authorized users.

## Background

Natural products have long been used as precious sources for the formulation of useful drugs (Cragg and Newman 2013; World Health Organization 2013; Veeresham 2012; Gyasi et al. 2011; Izzo and Ernst 2009; Kraft 2009). Besides combating diseases with the use of antibiotics, herbal medicines are also widely known for their implications in mitigating a number of clinical complications (Behera et al. 2013; Noor et al. 2013a; Tabassum and Ahmad 2011; Damery et al. 2011; Ernst 2011; Mendes et al. 2010). According to WHO estimation, 21,000 plants have been found globally to be used for medicinal purposes (Umashanker and Shruti 2011). Along with the progressive growth in synthetic medicines sourced from pharmaceuticals and biotech companies, medicinal plants have long been indeed readily available, reasonable and culturally suitable source of primary health care for the majority of Asia’s population (Srivastava et al. 1996).

An important shortfall of using antibiotics in recent days has appeared through the emergence of drug-resistant bacteria which in turn, resulted in the ineffectiveness of antibiotics (Dutta et al. 2013; Molton et al. 2013; Tenover 2006; Khan et al. 2013). Furthermore, a huge number of reports revealed the adverse effects and drug interactions of antibiotics resulting in fatal toxicity (Somasundaram and Manivannan 2013; Eric et al. 2008; Aster and Bougie 2007; Granowitz and Brown 2007; Apter et al. 2006; Navarro and Senior 2006; Baddour et al. 2005; Chow et al. 2005; Faulkner et al. 2005; Micek et al. 2005). Besides, when first-line and then second-line antibiotic treatment options get limited by the surfacing of bacterial drug-resistance traits or due to the drug-unavailability, physicians employing antibiotics appear to be eventually more toxic to the users. Patients with drug-resistant infections are often much more likely to expire, and even the survivors often need a longer hospital stays with prolonged healing. Earlier notation of anti-bacterial traits of natural products thus made an array of herbs to be the suitable alternative medication means of antibiotics (Barbour et al. 2004). Afterwards a number of reports revealed the antagonistic feature of herbs against a wide range of microorganisms as well as proved the lesser side effects with small or no toxicity of the herbal medicines compared to that of antibiotics (Djeussi et al. 2013; Kucekova et al. 2013; Oreagba et al. 2011; Kala et al. 2006).

In addition to the advantages over antibiotics, an important aspect is to be considered that herbs are more likely to harbor a huge number of bacteria including *Escherichia coli*, *Salmonella* spp., *Shigella* spp., aerobic spores, and the fungal population, usually originating in plantation soil or may be disseminated from organic fertilizer (Duraisankar and Ravindran 2013; Steven et al. 2013; World Health Organization 2007; Nakajima et al. 2005). Besides the method of harvesting herbs or herbal products, the production process, unhygienic handling, transportation and inappropriate storage may also cause microbial contamination as well as the chemical toxicity of the herbal medicines (World Health Organization 2007; Ting et al. 2013; Abba et al. 2009; Chan 2003).

In Bangladesh, a huge pharmacological evaluation and ethnomedicinal survey of medicinal plants have been conducted; however, the microbiological survey of herbal medicines have not been accomplished well (Das et al. 2012; Rahmatullah et al. 2010). While the research on microbiological assessment of the pharmaceutical products is abundant in this country, such work on the herbal products is quite petite (Das et al. 2013; Khanom et al. 2013; Noor et al. 2013a; Moniruzzaman et al. 2012; Hossain 2009). Along these lines, present study endeavored to assess microbiological contamination level within the commonly available herbal medicines; and to further demonstrate the anti-bacterial activity of the tested medicinal samples.

## Methods

### Study area, sampling and sample processing

The study was carried out with ten categories of oral herbal drugs in liquid formulations (details of the samples have been provided in the Additional file 1). Five (5) samples from each of the 10 categories were randomly collected from different drug’s store with appropriate dates of manufacturing and expiry within the city of Dhaka during the time frame of December 2013 to January 2014 according to the standard sampling method suggested by American Public Health Association (1998). All the samples were aseptically processed followed by homogenizing 10 ml of each samples with 90 ml normal saline and diluted up to 10^-6^ for the isolation and quantification of pathogenic bacteria and fungi.

### Microbiological analysis and confirmative biochemical tests

#### Estimation of total viable bacteria and fungi

For the enumeration of total viable bacteria (TVB) and the total fungal load, 0.1 mL of each sample was introduced onto the nutrient agar (NA) and Sabouraud’s dextrose agar (SDA) plates, respectively, by means of spread plate technique (Cappuccino and Sherman 1996). Plates were incubated at 37°C for 24 hours and at 25°C for 48 hours for total viable bacteria and fungi, respectively.

### Estimation of fecal coliform, *Escherichia coli*, *Klebsiella* spp*.*, *Staphylococcus* spp. and actinomycetes

From the dilutions 10^-3^ and 10^-5^, 0.1 mL of each sample was spread onto the membrane fecal coliform (MFC) agar and MacConkey agar for the enumeration of total fecal coliform (TFC), and coliforms (especially, *Escherichia coli* and *Klebsiella* spp.), respectively. Plates were incubated for 24 hours at 44.5°C and 37°C for fecal coliform and coliforms, correspondingly. Likewise, *Staphylococcus* spp. and actinomycetes were isolated onto Mannitol Salt Agar (MSA) and actinomycetes agar, respectively by adding 0.1 mL of diluted sample each, and all the plates were then incubated at 37°C for 24 hours.

### Isolation of *Salmonella spp., Shigella* spp. and *Vibrio* spp*.*

Ten (10) mL of sample was transferred into 90 ml of selenite cysteine broth (SCB) and alkaline peptone water (APW) for the enrichment of *Salmonella, Shigella*, and *vibrio* spp., respectively and incubated at 37°C for 6 hours. After incubation, the samples were diluted up to 10^-6^ and then 0.1 mL of samples from each of the 10^-3^ and 10^-5^ dilutions were spread onto *Salmonella-Shigella* (SS) agar and thiosulfate citrate bile salt sucrose (TCBS) agar for the isolation of *Salmonella* spp*. and Shigella* spp., *and Vibrio* spp., consecutively. Plates were incubated at 37°C for 48 hours for the detection of typical colonies. Finally, all the isolates were biochemically examined following standard procedures as described earlier (Cappuccino and Sherman 1996; Alfrad 2007).

### Antibiotic susceptibility test

Antibiotic susceptibility traits of the pathogenic isolates were examined (either drug resistant or sensitive) by the disc diffusion assay on Mueller-Hinton agar (MHA, Difco, Detroit, MI) against the commonly used antibiotics following the standard protocol (Munshi et al. 2012; Ferraro et al. 2001; Bauer et al. 1968). Commercially available laboratory grade antibiotic discs of Penicillin G (10 μg), Gentamicin (10 μg), Oxacillin (1 μg) Amoxicillin (30 μg), Imipenem (30 μg), Erythromycin (15 μg), Tetracycline (30 μg), Ciprofloxacin (5 μg),Trimethoprim-sulfamethoxazole (25 μg), Azithromycin (15 μg), Nalidixic acid (30 μg) and Ampicillin (10 μg) were aseptically placed over the surface of Mueller-Hinton agar plates at spatial distance of 5 mm which had been previously inoculated (with the afterward formation of bacterial lawn over the agar surface) with the pathogenic test bacterial suspensions with prescribed turbidity (compared to that of the McFarland standard of 0.5).

### Determination of anti-bacterial activity of herbal medicine

The anti-bacterial activity of the samples was performed by using agar well diffusion method (Ahmed et al. 2014; Jagessar et al. 2008; Hussain et al. 2010). At first, the suspensions (with standard turbidity compared to that of the McFarland standard of 0.5) of each of the test bacteria; i.e., *Pseudomonas* spp., *Listeria* spp., *Bacillus* spp., *Vibrio* spp., *Salmonella* spp., *Klebsiella* spp., *Staphylococcus* spp. and *E. coli* was spread evenly over the MHA using cotton swab which in turn resulted in the uniform lawns. Wells were then made spanning the MHA by means of sterile cork-borer. Each of the samples was then introduced separately in a concentration of 100 μL with a residual herbal medicine concentration of 2.4 mg/mL, 0.4 mg/mL, 0.6 mg/mL, 1 mg/mL, 3.5 mg/mL, 2.5 mg/mL, 3.1 mg/mL, 6.4 mg/mL, 0.9 mg/mL and 1.5 mg/mL for samples 1, 2, 3, 4, 5, 6, 7, 8, 9 and 10, consecutively in the specified well along with a positive control (Gentamicin, 10 μg) and a negative control (normal saline). Presence of clear zone around the sample solution (if any) was analytical for the existence of the antibacterial activity of the samples tested.

### Determination of Minimal Inhibitory Concentration (MIC)

Besides the agar well diffusion method, additionally the minimal inhibitory concentration (MIC) or broth microdilution assay was performed to determine the lowest concentration of each of the exoeriemntal herbel medicines capable of trimming down the extent of viability of the test bacteria (Carson et al. 1995). According to the suggested method by Clinical and Standared Laboratory Institute: two fold serial broth dillution method was used to determine the MIC (Clinical and Laboratory Standards Institute 2006). An aliquot of 100 μL of the ovenight (~12 hours) culture of each of the test bacteria was inoculated into the appropriately labeled sterile tubes containing Mueller Hinton (MH) broth (Oxoid Ltd, England) at the turbidity adjusted with 0.5 McFarland standard and the different volumes (32 μL, 64 μL, 128 μL, 256 μL, 512 μL, 1024 μL and 2048 μL) of herbal medicine samples were introduced onward to make a total volume of 3 ml. Then the residual or extract concentrations of the herbal medicines for each of the above mentioned volume of all the aqueous samples used in the MIC assay was determined (Additional file 2). All the tubes were incubated at 37°C for 24 hours. The least concentration (mg/mL) of each sample extract which could retard the multiplication of the tested bacteria, as judged visually by lack of turbidity in the tube comparable to the McFarland standard, was recorded and considered as the MIC value.

## Results and discussion

The practice and prospect of herbal medicines in Bangladesh is to its brim since the country has a number of operational traditional herbal medicinal systems including homeopathy, Ayurveda, Unani, folk medicine and home remedies (Akter et al. 2012; Das et al. 2012; Rahmatullah et al. 2010). The routine monitoring of the hygiene as well as the anti-bacterial potency of these drugs are thus required to ensure a sustainable health management system. To our knowledge, such examination of herbal medicinal samples is not that frequent in our country, which in turn, may pose the probable unidentified adverse toxicity upon usage of the medicines.

### Microbial prevalence within the herbal medicine samples

The microbial spoilage of herbal medicines is quite evident from our study where all the samples have been detected to harbour contaminant bacteria; nevertheless, all were indeed within the specified range of 10^3^-10^5^ cfu/mL (Table 1). However, contamination with the specific pathogenic bacterium *Staphylococcus aureus* (10^3^-10^5^ cfu/mL) in 8 samples were indicative of improper handling during harvesting, processing, manufacturing, distribution or storage of the medicinal plant samples (Duraisankar and Ravindran 2013; Steven et al. 2013; Duraisankar and Ravindran 2013, 2007; Ting et al. 2013; Abba et al. 2009; Chan 2003). Two of the samples tested were found to be contaminated with *Klebsiella* spp., which possibly might take place during the plant harvesting or even from the farming manure; and certainly due to the unhygienic handling (Duraisankar and Ravindran 2013; Steven et al. 2013; World Health Organization 2007). Interestingly the fungal species were quite infrequent and only one sample exhibited the fungal proliferation (Table 1).

**Table 1:** **Microbiological conditions of the samples (cfu/mL)**

Samples	TVB	Fungi	***Kebsiella*** spp.	FCC	***Staphylococcus*** spp.	Actinomycetes	***Salmonella*** spp.	***Shigella*** spp.	***Vibrio*** spp.
Sample 1 (N = 5)	6.5 × 10^5^	0	1.0 × 10^6^	0	4.0 × 10^4^	0	0	0	0
Sample 2 (N = 5)	7.0 × 10^5^	0	0	0	3.7 × 10^5^	0	0	0	0
Sample (N = 5)	2.4 × 10^4^	0	0	0	2.0 × 10^3^	0	0	0	0
Sample 4 (N = 5)	1.14 × 10^5^	0	0	0	4.6 × 10^4^	0	0	0	0
Sample 5 (N = 5)	9.0 × 10^4^	0	0	0	3.0 × 10^3^	0	0	0	0
Sample 6 (N = 5)	4.12 × 10^5^	0	0	0	2.1 × 10^4^	0	0	0	0
Sample 7 (N = 5)	1.06 × 10^5^	1.8 × 10^4^	4.5 × 10^4^	0	2.0 × 10^3^	0	0	0	0
Sample 8 (N = 5)	2.0 × 10^3^	0	0	0	0	0	0	0	0
Sample 9 (N = 5)	3.5 × 10^4^	0	0	0	6.0 × 10^2^	0	0	0	0
Sample 10 (N = 5)	7.7 × 10^3^	0	0	0	0	0	0	0	0

TVB: Total viable bacteria; FCC: fecal coliform count.

The average load has been shown.

Microbial limits (World Health Organization 2007).

Total aerobic bacteria 10^5^ cfu/ml.

*Escherichia coli* 10^1^ cfu/ml.

*Salmonella* spp*.* absent.

Enterobacteria 10^3^ cfu/mL.

### Drug-resistance trait of the pathogens isolated from herbal medicine samples

**Figure 1:**
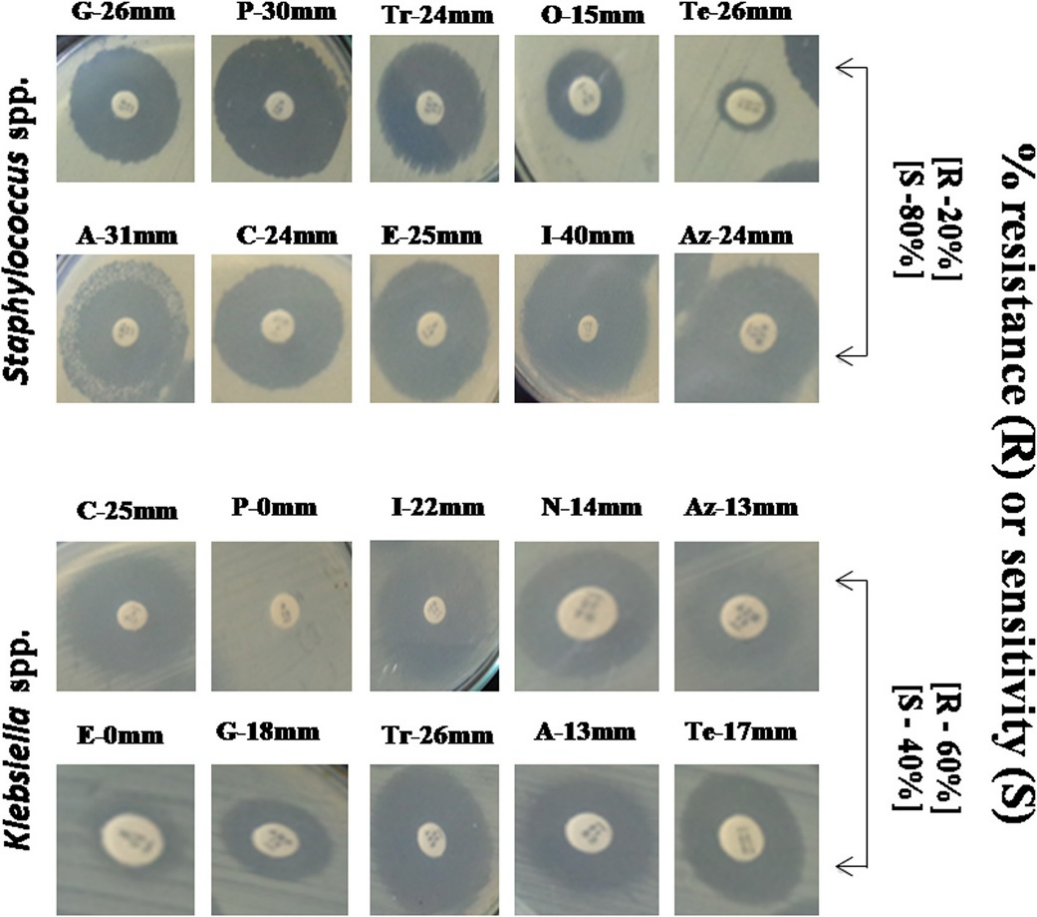
**Determination of resistance or susceptibility of** ***Staphylococcus*** **spp. and** ***Klebsiella*** **spp. towards commonly used antibiotics.** The arrows indicate the frequency of resistance/sensitivity of the isolates*. Khebsiella* spp. exhibited elevated resistance (60%) than *Staphylococcus* spp. (20%). P = Penicillin G (10 μg), G = Gentamicin (10 μg), O = Oxacillin (1 μg), A = Amoxicillin (30 μg), I = Imipeneme (30 μg), E = Erythromycin (15 μg), Te = Tetracycline (30 μg), C = Ciprofloxacin (5 μg), Tr = Trimethoprim-sulfamethoxazole (25 μg), Az = Azithromycin (15 μg), N = Nalidixic acid (30 μg), A = Ampicillin (10 μg), R: resistant, S: sensitive.

The surfacing of drug-resistant bacteria within an array of products (including water, food and pharmaceuticals) resulted in the incompetence of the synthetic drugs or antibiotics (Acharjee et al. 2013a, [b]; Ahmed et al. 2013; Dutta et al. 2013; Molton et al. 2013; Tenover 2006). Among the 2 pathogens found in our samples, *Klebsiella* spp. exhibited up to 60% resistance, and the pathogen was specifically found to be highly resistant against penicillin G and erythromycin; while conversely *S. aureus* was found to be sensitive towards most of the antibiotics used in this study (Figure 1). Nevertheless, existence of drug-resistant pathogenic isolate in 2 of the samples studied may pose further threat to the overall public health remedies through the application of these medicines.

Antimicrobial resistance is one of the most serious health threats. Clinical complications and infections from drug-resistant resistant bacteria are now frequently commencing, and surprisingly some pathogens have been identified to be resistant to multiple types or classes of antibiotics; i.e., multi-drug resistant (Dutta et al. 2013; Noor et al. 2013b). The thrashing of effectual antibiotics emasculates the capacity to fight against infectious diseases and gets along the infectious complications frequent in susceptible patients. Routine examination of herbal medicines for the presence of drug-resistant bacteria is thus obligatory.

### In vitro anti-bacterial activity of the herbal medicine samples

A number of reports showed the anti-bacterial features of natural herbs suggesting them appropriate substitute of synthetic medicines (Djeussi et al. 2013; Kucekova et al. 2013; Oreagba et al. 2011; Kala et al. 2006). However, the experimental demonstration of the existence of such anti-bacterial activity of the common and popular herbal medicines used by the Bangladeshi community is still lacking. Our study, for the first time, revealed such trait within our common herbal medicines whereby 70% of the tested samples were found to exhibit the in vitro anti-bacterial activity (Table 2). One of the samples (Sample no. 2) studied possessed the activity against almost all the test bacteria employed, one (Sample no. 5) was found to be effective against 5 test bacteria, one (Sample no. 6) against 3 test bacteria, 2 samples (Sample 3 and 9) against 2 test bacteria, while another 2 samples (Samples 1 and 10) exhibited the activity against only one bacterium. The anti-bacterial activity was found to be completely absent in 3 samples (Samples 4, 7 and 8).

**Table 2:** **Antimicrobial activity of the herbal medicine samples tested**

Herbal medicine samples (concentration in mg/mL)	Zone of inhibition (mm) against test bacteria							
	***E. coli***	***Klebsiella*** spp.	***Salmonella*** spp.	***Vibrio*** spp.	***Pseudomonas*** spp.	***Bacillus*** spp.	***Staphylococcus*** spp.	***Listeria*** spp.
Sample 1 (2.4)	0	0	0	0	0	0	0	11 mm
Sample 2 (0.4)	11 mm	15 mm	20 mm	20 mm	17 mm	20 mm	18 mm	20 mm
Sample 3 (0.6)	0	11 mm	0	0	0	0	0	7 mm
Sample 4 (1.0)	0	0	0	0	0	0	0	0
Sample 5 (3.5)	0	18 mm	0	10 mm	11 mm	10 mm	0	10 mm
Sample 6 (2.5)	0	16 mm	0	0	0	8 mm	0	9 mm
Sample 7 (3.1)	0	0	0	0	0	0	0	0
Sample 8 (6.4)	0	0	0	0	0	0	0	0
Sample 9 (0.9)	0	11 mm	0	13.9 mm	0	0	0	0
Sample 10 (1.5)	0	0	0	12 mm	0	0	0	0

The experiments were conducted three times independently, and the results were found to be reproducible. One representative data has been shown.

In addition, the in vitro anti-bacterial activity of the samples was further supported by observing the result of MIC (Table 3). In this parameter all the samples were found to show their anti-bacterial activity against different tested bacteria; i.e., *E. coli, Klebsiella* spp., *Pseudomonas* spp., *Bacillus* spp., *Salmonella* spp., *Listeria* spp., *Staphylococcus* spp., and *Vibrio* spp. The highest MIC was scored at 66 mg/mL while the lowest value was noted to be 1 mg/mL (Table 3). For sample 1, the ideal MIC was found to be 25 mg/mL against all the tested pathogenic bacteria except *Pseudomonas* spp. which was found to be inhibited at a concentration of 50 mg/mL. Sample 2 revealed the MIC at a maximal level of 7 mg/mL to stall the bacterial growth. For sample 3, the MIC was recorded to be not more than 6 mg/mL to inhibit any of the tested bacterial growth. Sample 4 showed the MIC potential at 10 mg/mL against most of the tested pathogenic bacteria while 5 mg/mL and 20 mg/mL were found to be effective against *E. coli* and *Listeria* spp., respectively. Sample 5 exhibited the MIC range between 18 mg/mL to 36 mg/mL to inhibit the bacterial growth. In case of sample 6, 13 mg/mL was effectual to hinder most of the test bacterial growth excluding *Listeria* spp. and *Vibrio* spp. In case of sample 7, the MIC was found at 16 mg/mL against *E. coli*, *Klebseilla* spp. *Bacillus* spp., 50 mg/mL against *Pseudomonas* spp., *Salmonella* spp., *Staphylococcus* spp., *Vibrio* spp., and 66 mg/mL against *Listeria* spp. For sample 8, the MIC was recorded at 66 mg/mL against most of the bacterial species except *E. coli* and *Staphylococcus* spp. (with an MIC of 33 mg/mL). For samples 9 and 10, the maximal MICs were determined to 18 mg/mL and 30 mg/mL, respectively (Table 3).

**Table 3:** **Minimum Inhibitory Concentration (MIC) of the samples**

Sample	Organisms							
	***E. coli***	***Klebsiella*** spp.	***Salmonella*** spp.	***Vibrio*** spp.	***Pseudomonas*** spp.	***Bacillus*** spp.	***Staphylococcus*** spp.	***Listeria*** sppcm
Sample 1	25 mg/mL	25 mg/mL	25 mg/mL	25 mg/mL	51 mg/mL	25 mg/mL	25 mg/mL	25 mg/mL
Sample 2	1 mg/mL	3 mg/mL	3 mg/mL	3 mg/mL	7 mg/mL	7 mg/mL	3 mg/mL	7 mg/mL
Sample 3	3 mg/mL	1 mg/mL	3 mg/mL	3 mg/mL	6 mg/mL	3 mg/mL	6 mg/mL	6 mg/mL
Sample 4	5 mg/mL	10 mg/mL	10 mg/mL	10 mg/mL	10 mg/mL	10 mg/mL	10 mg/mL	20 mg/mL
Sample 5	18 mg/mL	36 mg/mL	36 mg/mL	36 mg/mL	36 mg/mL	18 mg mL	18 mg/mL	36 mg/mL
Sample 6	13 mg/mL	13 mg/mL	13 mg/mL	26 mg/mL	13 mg/mL	13 mg/mL	13 mg/mL	26 mg/mL
Sample 7	16 mg/mL	16 mg/mL	50 mg/mL	50 mg/mL	50 mg/mL	16 mg/mL	50 mg/mL	66 mg/mL
Sample 8	33 mg/mL	66 mg/mL	66 mg/mL	66 mg/mL	66 mg/mL	66 mg/mL	33 mg/mL	66 mg/mL
Sample 9	9 mg/mL	18 mg/mL	9 mg/mL	9 mg/mL	9 mg/mL	18 mg/mL	9 mg/mL	18 mg/mL
Sample 10	15 mg/mL	15 mg/mL	15 mg/mL	15 mg/mL	30 mg/mL	7 mg/mL	30 mg/mL	30 mg/mL

Residual concentrations of the herbal medicines have been provided.

The experiments were conducted three times independently, and the results were found to be reproducible. One representative data has been shown.

However, agar well diffusion test may not be a suitable one to determine the antibacterial activity of natural compounds. The rate of diffusion of natural antimicrobials can be strongly affected by the polarity, the concentration, the molecular size, etc. of the compounds. This fact was evident from our study findings (Tables 2 and 3). When the results of agar well diffusion method and MIC assay were compared, a clear discrepancy was apparent for some samples. Although a relatively low concentration of sample 3 (up to 6 mg/mL) was found to inhibit the growth of all the tested bacteria as revealed from the MIC assay, only *Klebsiella* spp. and *Listeria* spp. were found to be affected by the concentration of 0.6 mg/mL in agar well diffusion method with considerably small zone of inhibition. Similar divergence was noticed for the samples 4, 9 and 10. Additionally, sample 4 exhibited no anti-bacterial activity in agar well diffusion method, whereas the sample was found to possess the MIC level of 10 mg/mL for most of the test bacteria. Sample 9 featured MIC of 9 mg/mL against *Pseudomonas* spp., *Salmonella* spp., *Staphylococcus* spp. and *Vibrio* spp., but neither of the bacteria except *Vibrio* spp. were found to be inhibited in the agar well diffusion method. Sample 10 exhibited an MIC of 7 mg/mL against *Bacillus* spp. which was conversely found to be unaffected in the agar well diffusion assay.

Samples 1, 2, 7 and 8 on the other hand showed nearly consistent results as noticed both from the agar well diffusion assay and the broth microdilution assay. Sample 2 had minimal MIC as sample 3 and exhibited the anti-bacterial activity against all the tested bacteria as was also noticed in the agar well diffusion assay. Samples 1, 7 and 8 had almost no antibacterial activity and consistently featured moderate to high MIC. Exceptionally, Sample 5 imparting a relatively moderate MIC showed anti-bacterial activity against most of the bacteria. Considering this exception it can be concluded that agar well diffusion method could be used as screening test but however, more reliable results could be achieved by the minimal inhibitory concentration (MIC); i.e., the broth microdilution assay.

## Conclusion

The revelation of the drug-resistant microbial pathogenic contaminants in the herbal medicines tested in our study is suggestive of implementing more hygienic manufacturing and processing operations. Nevertheless, the total viable bacteria were more or less with the specification limits which providentially ensured the overall product quality. The anti-bacterial traits of the samples tested revealed the potency of the medicines against pathogenic bacteria as well as the biological mechanism of action of such natural medicines. However, as revealed from the agar well diffusion tests, in most of the cases, the absence of such activity further urges for a careful formulation of the medicinal products to achieve the treatment efficiency against microbial pathogens. Besides, exploration of the motivating factors for the farmers cultivating medicinal plants as well as expanding the market of herbal medicinal plants in Bangladesh and in other developing countries would further aid in a better management of the overall public health.

## Authors’ information

All authors are from Department of Microbiology, Stamford University Bangladesh. MS and ITN is the thesis students of MS program of the department. MA and SK are working as Lecturer. RN, corresponding author of the manuscript, is the Departmental Head.

## Electronic supplementary material

Additional file 1: Names, composition and indications of the herbal medicine samples studied.(DOC 98 KB)

Additional file 2: Residual or extract concentrations of herbal medicines for each volume of aqueous samples used in the minimum inhibitory concentration (MIC) assay.(DOC 34 KB)
