# Supplementary material for: Microbiological profiling and the demonstration of in vitro anti-bacterial traits of the major oral herbal medicines used in Dhaka Metropolis
Source: Springerplus. 2014 Dec 15;3:739. doi: 10.1186/2193-1801-3-739 (PMC4320176; doi:10.1186/2193-1801-3-739)
Supplement: Supplementary file 1 — Additional file 1: Names, composition and indications of the herbal medicine samples studied.(DOC 98 KB) [file 40064_2014_1495_MOESM1_ESM.doc]

**Additional file 1** Names, composition and indications of the herbal medicine samples studied

| **Sample 1: Saduri (Hamdard)** | | | |
| --- | --- | --- | --- |
| Composition: Each 5ml contains (Aqueous Extract) | | | Indications:  Catarrh .  Cough.  Whooping cough.  Asthma.  Breathing discomfort.  Bronchial debility. |
| *Zizyphus vulgaris* | 100 mg | |
| *Sisymbriumirio* | 100 mg | |
| *Hyssopusofficinalis* | 93.50 mg | |
| *Ocimum sanctum* | 50 mg | |
| *Adhatodavasica* | 26.50 mg | |
| *Ephedra vulgaris* | 7.50 mg | |
| **Sample 2: Alkuli (Hamdard)** | | | |
| Composition: Each 5ml contains (Aqueous Extract) | | | Indications:  Pyrexia Amenorrhoea  AnnuriaHepatitis  OliguriaObstructive jaundice.  It is also very effective to clear the morbid substances from the kidney and urinary bladder. |
| *Cichoriumendivia* (root) | | 250 mg |
| *Cucumismelo* (seed) | | 250 mg |
| *Foeniculumvulgare* (root) | | 125mg |
| *Foeniculumvulgare* (seed) | | 125 mg |
| *Cichoriumendivia* (seed) | | 125 mg |
| *Tribulusterrestris* | | 125mg |
| **Sample 3:Fevnil (Hamdard)** | | | |
| Composition: Each 5ml contains (Aqueous Extract) | | | Indications:  Fever  Measles  Chicken Pox  Typhoid |
| *Sisymbriumirio* | 0.25 g | |
| *Zizyph us jujuba* | 0.25 g | |
| *Foeniculumvulgare* | 0.25 g | |
| *Boragoofficinalis* | 0.15g | |
| **Sample 4:Alvasin (Hamdard)** | | | |
| Composition: Each 5 ml syrup contains (Aqueous Extract) | | | Indications: |
| *Adhatodavasica*(leaf) | 1.00 g | | Cold |
| *Cordiadichotoma* | 0.20 g | | Dry Cough |
| *Glycyrrhizaglabra* | 0.05 g | | Congestion of Lugs |
| *Althaeaofficinalis*(seed) | 0.05 g | |  |
| *Malvasylvestris*(seed) | 0.05 g | |
| *Nymphaea nouchali* | 0.05 g | |
| *Centellaasiatica* | 0.05 g | |
| *Cydoniaoblonga*(seed) | 0.05 g | |
| **Sample 5: Carmina (Hamdard Unani Products)** | | | |
| Composition: Each 5ml contains (Aqueous Extract) | | | Indications: |
| *Piper nigrum* | 150 mg | | Anorexia |
| *Citrus aurantifolia* | 100 mg | | Indigestion |
| *Trachyspermumammi* | 100 mg | | Hyperacidity |
| *Cinnamomumzeylanicum* | 50 mg | | Flatulence |
| *Emblicaofficinalis* | 50 mg | | Constipation |
| *Terminaliabellerica* | 38 mg | | Abdominal pain due to gases |
| *Terminaliachebula* | 38 mg | | Stomach and liver disorders |
| *Zingiberofficinale* | 10 mg | |
| Sea salt | 8 mg | |

| **Sample 6: Jernide (Hamdard)** | | |
| --- | --- | --- |
| Composition: Each 5ml contains (Aqueous Extract) |  | Indications:  Spermatorrhoea Nocturnal emission & enuresis  Painful micturations |
| *Glycyrrhizaglabra* | 300 mg |
| *Cascara sagrada* | 100 mg |
| Tincture Belladona | 0.15 ml |
| **Sample 7: Naunehal (Hamdard)** | | |
| Composition: Each 5ml contains (Aqueous Extract) | | Indications: |
| *Pimpinellaanisum* | 2.50 mg | For digestive disorders and teething troubles |
| *Anithumsowa* | 2.50 mg | It is a palatable way to fight against griping |
| *Amomumsubulatum* | 2.50 mg | Flatulence |
| *Menthaarvensis* | 2.50 mg | Convulsion |
| *Hordeumvulgare* | 40 mg | Diarrhea and indigestion |
| Oil Anise | 0.0035 ml | So common among infants and teething children |
| **Sample 8: Santara-Jayson (Jayson natural products)** | | |
| Composition: Each 5ml contains (Aqueous Extract) | | Indications:  Oliguria  Urethritis  Painful micturation  Urinary tract infections  Febrile conditions |
| *Tribulusterrestris* | 200 mg |
| *Foeniculumvulgare roots* | 150 mg |
| *Foeniculumvulgare seeds* | 150 mg |
| *Nymphaea alba* | 150 mg |
| *Citrus reticulata* | 250 mg |
| *Citrus aurantifolia* | 75 mg |
| **Sample 9: Adovas (Saquare)** | | |
| Composition: Each 5ml contains (Aqueous Extract) | | Indications: |
| *Adhatodavasica* | 0.68 gm | Relieves cough |
| *Piper longum* | 0.14 gm | Soothes & liquefies phlegm |
| *Glycyrrhizaglabra* | 6.78 mg | Effective in smoker’s cough |
| *Zingiberofficinale* | 6.78 mg | Relieves sore throat |
| *Piper nigrum* | 6.78 mg |  |
| *Terminaliachebula* | 73.24 mg |
| *Saussurealappa* | 6.78 mg |
| *Syzgiumaromaticum* | 6.78 mg |
| *Eletteriacardamomum* | 6.78 mg |
| *Cinnamomumzeylanicum* | 6.78 mg |
| *Cinnamomumtamala* | 6.78 mg |
| *Pistaciaintegerrima* | 6.78 mg |
| *Myricanagi* | 6.78 mg |
| **Sample 10: Hepatolin (ACME)** | | |
| Composition: Each 5ml contains (Aqueous Extract) | | Indications: |
| *Aphanamixispolystachya* | 1.52 gm | Jaundice |
| *Woodfordiafructicosa* | 0.24 gm | Toxic liver injury |
| *Emblicaofficinalis* | 15.24 mg | Hepatitis |
| *Terminaliachebula* | 15.24 mg | Chronic indigestion |
| *Terminaliabelerica* | 15.24 mg | Anorexia |
| *Zingiberofficinale* | 15.24 mg |  |
| *Piper longum*(seed) | 15.24 mg |
| *Piper longum*(root) | 15.24 mg |
| *Elettariacardamomum* | 15.24 mg |
| *Cinnamomumtamala* | 15.24 mg |
