# Supplementary material for: Microbiological profiling and the demonstration of in vitro anti-bacterial traits of the major oral herbal medicines used in Dhaka Metropolis
Source: Springerplus. 2014 Dec 15;3:739. doi: 10.1186/2193-1801-3-739 (PMC4320176; doi:10.1186/2193-1801-3-739)
Supplement: Supplementary file 2 — Additional file 2: Residual or extract concentrations of herbal medicines for each volume of aqueous samples used in the minimum inhibitory concentration (MIC) assay.(DOC 34 KB) [file 40064_2014_1495_MOESM2_ESM.doc]

**Additional file 2 Residual or extract concentrations of herbal medicines for each volume of aqueous samples used in the minimum inhibitory concentration (MIC) assay**

| **Volume of the sample used (µL)** | **Residual concentration of herbal medicine (mg/mL)** | | | | | | | | | |
| --- | --- | --- | --- | --- | --- | --- | --- | --- | --- | --- |
|  | **Sample 1** | **Sample 2** | **Sample 3** | **Sample 4** | **Sample 5** | **Sample 6** | **Sample 7** | **Sample 8** | **Sample 9** | **Sample 10** |
| **32** | 0.8 | 0.1 | 0.2 | 0.3 | 1.1 | 0.8 | 1.0 | 2.1 | 0.3 | 0.5 |
| **64** | 1.6 | 0.3 | 0.4 | 0.6 | 2.3 | 1.6 | 2.0 | 4.1 | 0.6 | 0.9 |
| **128** | 3.1 | 0.5 | 0.8 | 1.3 | 4.6 | 3.3 | 4.1 | 8.3 | 1.2 | 1.9 |
| **256** | 6.3 | 1.0 | 1.6 | 2.5 | 9.1 | 6.6 | 8.2 | 16.5 | 2.3 | 3.8 |
| **512** | 12.5 | 2.0 | 3.2 | 5 | 18.2 | 13.1 | 16.3 | 32.9 | 4.6 | 7.6 |
| **1024** | 25.0 | 4.1 | 6.4 | 10 | 36.4 | 26.2 | 32.6 | 65.8 | 9.2 | 15.1 |
| **2048** | 50.0 | 8.2 | 12.7 | 20 | 72.8 | 52.4 | 65.2 | 131.6 | 18.4 | 30.2 |
